# Predicting development of ipilimumab-induced hypophysitis: utility of T4 and TSH index but not TSH

**DOI:** 10.1007/s40618-020-01297-3

**Published:** 2020-05-24

**Authors:** M. S. Siddiqui, Z. M. Lai, L. Spain, V. Greener, S. Turajlic, J. Larkin, D. L. Morganstein

**Affiliations:** 1 Department of Endocrinology, Chelsea and Westminster Hospital, 369 Fulham Road, London, SW10 9NH UK; 2 Skin Unit, Royal Marsden Hospital, London, UK

**Keywords:** Hypophysitis, Melanoma, Ipilimumab, TSH index

## Abstract

**Purpose:**

Ipilimumab, a monoclonal antibody inhibiting CLTA-4, is an established treatment in metastatic melanoma, either alone or in combination with nivolumab, and results in immune mediated adverse events, including endocrinopathy. Hypophysitis is one of the most common endocrine abnormalities. An early recognition of hypophysitis may prevent life threatening consequences of hypopituitarism; therefore, biomarkers to predict which patients will develop hypophysitis would have clinical utility. Recent studies suggested that a decline in TSH may serve as an early marker of IH. This study was aimed at assessing the utility of thyroid function tests in predicting development of hypophysitis.

**Methods:**

A retrospective cohort study was performed for all patients (*n* = 308) treated with ipilimumab either as a monotherapy or in combination with nivolumab for advanced melanoma at the Royal Marsden Hospital from 2010 to 2016. Thyroid function tests, other pituitary function tests and Pituitary MRIs were used to identify those with hypophysitis.

**Results and conclusions:**

Ipilimumab-induced hypophysitis (IH) was diagnosed in 25 patients (8.15%). A decline in TSH was observed in hypophysitis cohort during the first three cycles but it did not reach statistical significance (*P* = 0.053). A significant fall in FT4 (*P* < 0.001), TSH index (*P* < 0.001) and standardised TSH index (*P* < 0.001) prior to cycles 3 and 4 in hypophysitis cohort was observed. TSH is not useful in predicting development of IH. FT4, TSH index and standardised TSH index may be valuable but a high index of clinical suspicion remains paramount in early detection of hypophysitis.

**Electronic supplementary material:**

The online version of this article (10.1007/s40618-020-01297-3) contains supplementary material, which is available to authorized users.

## Introduction

Advanced malignant melanoma had historically poor prognosis and a median survival of only 6 to 9 months before the availability of immunotherapy [1]. The introduction of immunotherapy using immune checkpoint inhibitors (ICPIs) and targeted therapy has shown remarkable improvement in progression-free and overall survival compared with chemotherapy. Ipilimumab is a humanized monoclonal antibody that acts as an inhibitor of cytotoxic T-lymphocyte-associated protein 4 (CTLA-4), which in turn leads to activation of T cells resulting in tumour cell death. Ipilimumab was the first drug in a randomised trial to show a survival benefit in patients with advanced melanoma [2]. A primary analysis of overall survival (OS) data from 12 ipilimumab phase II and phase III trials calculated a median OS of 11.4 months and 3-year survival rates of 22%.

Ipilimumab is licensed in melanoma either alone, or in combination with nivolumab, a fully human IgG4 monoclonal antibody that inhibits programmed cell death (PD-1) receptor. PD-1 receptors inhibit T cell activation by binding to ligands PD-L1 and PD-L2 which are expressed on antigen-presenting cells and 40–50% of melanomas [3]. Nivolumab increases T cell activation and subsequent anti-tumour activity. Combined administration with ipilimumab and nivolumab has demonstrated higher level of efficacy but is associated with increased toxicity [4, 5] and has now become standard of care.

Increasing use of ICPIs has led to a rise in a unique spectrum of side effects known as immune-related adverse events (irAEs), thought to be result of impaired self-tolerance from increased T cell activation [6], with the most common manifestations in dermatologic, gastrointestinal, hepatic and endocrine systems.

A systematic review and meta-analysis of 38 randomised controlled trials and 7551 patients estimated a 10 percent overall incidence of clinically significant endocrinopathies, in patients treated with ICPIs [7]. These include autoimmune thyroiditis, hypophysitis, adrenal insufficiency and immune mediated diabetes.

Ipilimumab induced hypophysitis (IH) is amongst the most commonly reported endocrine irAEs following ipilimumab treatment, with an incidence of 3.9–13.3% frequently resulting in deficiencies of multiple pituitary axes. The most common clinical presentation includes headache and fatigue, sometimes with pituitary enlargement [8, 9]. In animal models, inflammatory infiltrates of macrophages and lymphocytes were observed in focal areas of pituitary in response to anti-CTLA4 antibody injections [10]. Histological features in man have not been ascertained as patients have not required surgery. Diagnosis is, therefore, established by clinical presentation, biochemical evidence of pituitary hormone deficiencies, whilst pituitary enlargement with enhancement on MRI is sometimes observed. Early detection may prevent potentially life-threatening consequences of hypopituitarism, especially adrenal insufficiency. Diagnosis can be challenging due to the nonspecific nature of the symptoms, especially on a background of advanced malignancy, whilst MRI findings are not universal. Therefore, clinical and biochemical predictors of hypophysitis would be helpful in allowing prompt treatment with hormone replacement.

In advanced melanoma, the recommended dose of ipilimumab is 3 mg/kg administered intravenously; this continues every 3 weeks for a total of four doses. Each 3-week period constitutes a ‘treatment cycle’. The summary of product characteristics (SPC) of ipilimumab recommends evaluation of thyroid function tests (TFTs) before each treatment cycle. A decline in thyroid stimulating hormone (TSH) has been suggested as a possible predictive factor for the development of ipilimumab-induced hypophysitis [11]. De Sousa et al. in a recent study suggested that a fall in TSH ≥ 80% may be an early predictor of IH. However, this was a small study (only 9 patients) and the criteria for diagnosis of hypophysitis were not clear (for example one of the hypophysitis patients was already on corticosteroid treatment at diagnosis so could not have assessment of ACTH function) [12].

Jostel et al. proposed the TSH index (TSHI) ‘a Free T4 adjusted TSH’ as a possible predictor of secondary hypothyroidism, whilst the thyroid functions were still within the reference ranges. A second standardised form of TSHI was based on mean values (2.7) and standard deviations (0.676) of TSHI and was calculated as (TSHI—2.70)/0.676 [13]. This study assesses the utility of thyroid hormone levels including the TSH index and standardised TSH index to predict the development of IH.

## Patients and methods

### Subjects

The study identified all patients treated with ipilimumab therapy as either monotherapy or in combination with nivolumab for advanced melanoma at The Royal Marsden Hospital (London, United Kingdom) between 13-September-2010 and 31-December-2015 for monotherapy, and between 5-August-2013 and 13-January-2016 for combination therapy. 308 patients were identified, consisting of 277 ipilimumab monotherapy patients and 31 combination therapy patients. Patients were scheduled to receive a maximum of four doses of ipilimumab, once every 3 weeks. Patients enrolled in ongoing clinical trials at the time of analysis were excluded from the study.

Patient data for this study was retrospectively gathered from electronic medical records. Data was collected on patient characteristics, clinical presentation, radiological imaging, TFT results, and testing of other pituitary axes.

### Biochemical testing

TSH, FT_4_ and FT_3_ were measured using an Abbott Architect method in all patients at baseline and at the start of each treatment cycle. In cases, where there were multiple undetectable TSH readings (< 0.03 mIU/l), the TSH reading with the lowest FT_4_ level was taken as the nadir TSH level. In calculation of TSH index (TSHI) and standardised TSHI, 2 patients with TSH falling to undetectable levels (< 0.03 mIU/l) were imputed as 0.03 mIU/l. Cortisol was measured with an Abbott Architect method.

### Radiological investigation and pituitary axis testing

Pituitary MRIs were performed according to clinical indication such as a new onset headache. Patients with suspected hypophysitis also received pituitary axis testing according to clinical indication. The blood tests included luteinising hormone (LH), follicle-stimulating hormone (FSH), testosterone, ACTH, cortisol, insulin-like growth factor 1 (IGF-1), growth hormone and prolactin.

The diagnosis of hypophysitis was based on the presence of one or more of the following criteria (adapted from [14]:

1. Secondary hypothyroidism. Defined as a low free T4 level (< 9.1 pmol/l) with a normal or suppressed TSH level that did not normalise on further testing.
2. Secondary adrenal insufficiency. Defined as either A) a low random cortisol level of < 100 nmol/l with a non-elevated ACTH level in the absence of exogenous steroids, or B) a random cortisol of < 150 nmol/l with symptoms of adrenal insufficiency (fatigue, nausea, weight loss) in the presence of at least 1 other pituitary hormone deficiency or enlarged pituitary on MRI.
3. A MRI showing diffuse enlargement and/or abnormal enhancement of the pituitary gland which subsequently resolves.

Two definitions of secondary adrenal insufficiency were used as ACTH levels were not investigated in all patients due to the acute presentation in some. Although not used for diagnostic purposes, information on the gonadal axis and prolactin levels were also collected. Growth hormone axis was not formally assessed. The exact date at which hypophysitis developed can be difficult to determine as patients may not be immediately symptomatic. In this study, the date of hypophysitis was taken as either the date at which the patient first experienced a headache or the date of the first abnormal TSH result, whichever occurred first.

Patients with known primary hypothyroidism or on levothyroxine therapy prior to initiation of ipilimumab were excluded from the study. In addition patients who developed an elevated TSH during treatment, suggesting primary or sub-clinical hypothyroidism were also excluded from the analysis, although those who developed thyroid abnormalities after the 4 cycles of ipilimumab were administered were not excluded, as only thyroid hormone levels during treatment were analysed in this study.

Thyroid function result at each cycle of ipilimumab treatment were collected from the Royal Marsden EPR, and were compared between baseline and each cycle in those with and without hypophysitis, as well as between groups at each cycle.

TSH, free T4, percentage change in TSH from baseline, TSH Index, standardised TSH index prior to each cycle of ipilimumab treatment were evaluated to determine their ability to predict development of hypophysitis.

### Statistical techniques

Descriptive statistics are presented as mean ± standard deviation or medians ± interquartile range. As not all results were normally distributed, values were compared with Kruskal–Wallis test with Dunn’s post-hoc analysis between groups. ROC analysis was used to delineate area under the curve, sensitivity and specificity of related values. Statistical significance was defined as *p* < 0.05. All analyses were conducted using GraphPad Prism version 5.01 for Windows (GraphPad Software, La Jolla California USA).

## Results

### Patient characteristics

The cohort consisted of 308 ipilumumab treated patients; 277 patients received ipilimumab alone and 31 received ipilimumab and nivolumab in combination. 25 patients (8.11%) were identified to have ipilimumab-induced hypophysitis.

The median age of the hypophysitis cohort was older at 65 (range 32 to 79) years compared to 58 (range 16 to 88) years for the no hypophysitis cohort but this did not reach significance (*p* = 0.053). There was no significant difference in gender ratios (*p* = 0.71) (12 M and 13 F). Median follow up (defined as time from first cycle of ipilimumab to last date of contact or death) was 223 days (IQR 112–419).

Patients who developed hypophysitis received a median of 3 (range 3–4) treatment cycles of ipilimumab, whilst patients who did not develop hypophysitis received a median of 4 (range 2–4) treatment cycles.

### Clinical presentation

Of the 25 patents with hypophysitis, 22 patients experienced headaches, whilst 3 patients did not; the mean time from treatment initiation to onset of headache was 58.4 (± 24.4) days. Two patients had visual field defects unrelated to hypophysitis, one due to brain metastases and the other had a long-standing visual field defect, predating treatment. 21 patients reported fatigue and the mean time from treatment initiation to onset of fatigue was 65.8 (± 40.4) days.

### Radiological findings

24 out of 25 patients with hypophysitis underwent pituitary MRIs; 12 had pituitary gland enlargement, whilst 12 had a normal pituitary gland. All three patients without a headache had normal MRIs. The median time from onset of headache to MRI was 1.5 days in those, where the pituitary was enlarged and 7 days, where it was normal, but this was not significant *(p* = 0.11).

### Thyroid function tests

At baseline, 22 out of 25 patients with hypophysitis had normal thyroid function. One patient did not have baseline thyroid function test. Two patients had low TSH levels of 0.04 mIU/l and 0.43 mIU/l, respectively, with FT_4_ levels within reference range at baseline; one of whom later developed secondary hypothyroidism. 11 patients were noted to have a decline in TSH level below the reference range (1 patient before cycle 2, 7 patients before cycle 3, 3 patients before cycle 4). Out of these 11 patients, only 7 subsequently developed secondary hypothyroidism based on a free T4 below the reference range. Based on the study criteria, secondary hypothyroidism was diagnosed in 17 patients giving an incidence rate of 68.0%.

Of the controls, thyroid function was not available at baseline in 49 patients, whilst 17 patients developed a rise in TSH above the reference range suggestive of subclinical or primary hypothyroidism and were excluded from the analysis, leaving 217 controls in the thyroid function analysis. One patient in the hypophysitis group developed co-existing sub-clinical primary hypothyroidism after the treatment period, which was not an exclusion criterion.

### Other pituitary axes

**Fig. 1:**
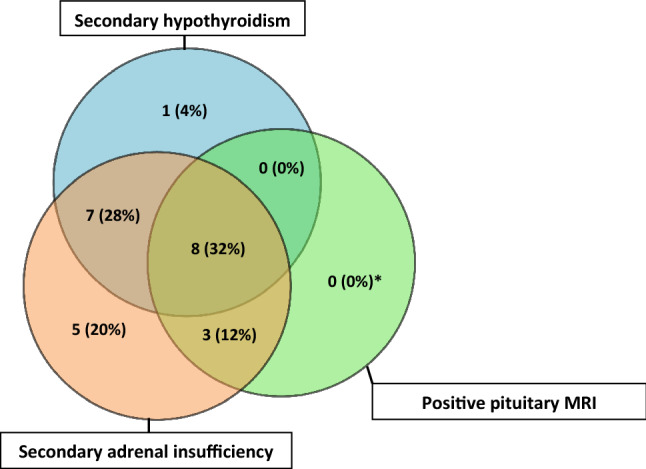
Venn diagram showing the proportions of the three common abnormalities in ipilimumab-induced hypophysitis (Total cases *n* = 25; *one patient did not have MRI)

24 out of 25 patients developed secondary adrenal insufficiency. 7 out of 13 men had secondary hypogonadism (one was on finasteride and, therefore, was not evaluated), whilst 8 out of 12 women had either secondary hypogonadism or inappropriately low or normal gonadotrophin levels for a post-menopausal state (with LH more commonly involved than FSH). 12 patients had low prolactin levels, whilst 1 had elevated prolactin levels at the time of diagnosis. Only 4 patients had single axis affected, and in all cases this was secondary adrenal insufficiency. Clinical details of the patients are presented in Supplementary Table 1, and a summary of the findings leading to diagnosis of hypophysitis are shown in Fig. 1.

### Thyroid function at each treatment cycle

Thyroid function was available at baseline in 24 patients in the hypophysitis group, and 217 controls. Prior to cycle 2, data was available for 19 and 168 patients and controls, respectively, and at cycle 3, this was 17 and 117 and at cycle 4, 10 and 86. This was because not all patients received all 4 cycles of treatment due to other irAEs or deaths. Mean values of TSH, Free T4, TSH Index, Standardised TSH index and percentage change in TSH from pre-cycle 1 levels were compared between normal and patients with hypophysitis at each cycle, and the diagnostic performance to detect hypophysitis determined at cycle 3 for each part of thyroid function. Thus the seven patients who were diagnosed with hypophysitis before the third cycle of ipilimumab were excluded from further analysis, so all remaining analysis is based on thyroid function tests at the third cycle of ipilimumab, prior to the diagnosis of hypophysitis. Patients included at each stage are summarised in Supplementary Fig. 1. Thyroid indices prior to each cycle are summarised in Table 1.

**Table 1:** Median values of thyroid indices prior to each cycle of treatment in controls and those who subsequently developed ipilimumab hypophysitis (IH)

	Cycle 1		Cycle 2		Cycle 3		Cycle 4	
	Control	IH	Control	IH	Control	IH	Control	IH
TSH (mU/L)	1.490	1.375	1.455	1.160	1.360	0.4800	1.445	0.3400
T4 (pmol/L)	13.40	12.25	13.50	13.90	14.10	11.00***	13.85	10.70*
TSH Index	1.980	1.840	2.000	1.790	2.010	1.140***	2.015	0.6050*
sTSH Index	− 1.070	− 1.275	− 1.035	− 1.340	− 1.020	− 2.310***	− 1.015	− 3.100*
% Change in TSH			− 5.850	− 9.420	− 6.830	− 41.00	− 4.840	− 81.52

*P, 0.05 between controls and IH at that cycle

***P < 0.001

### TSH

**Fig. 2:**
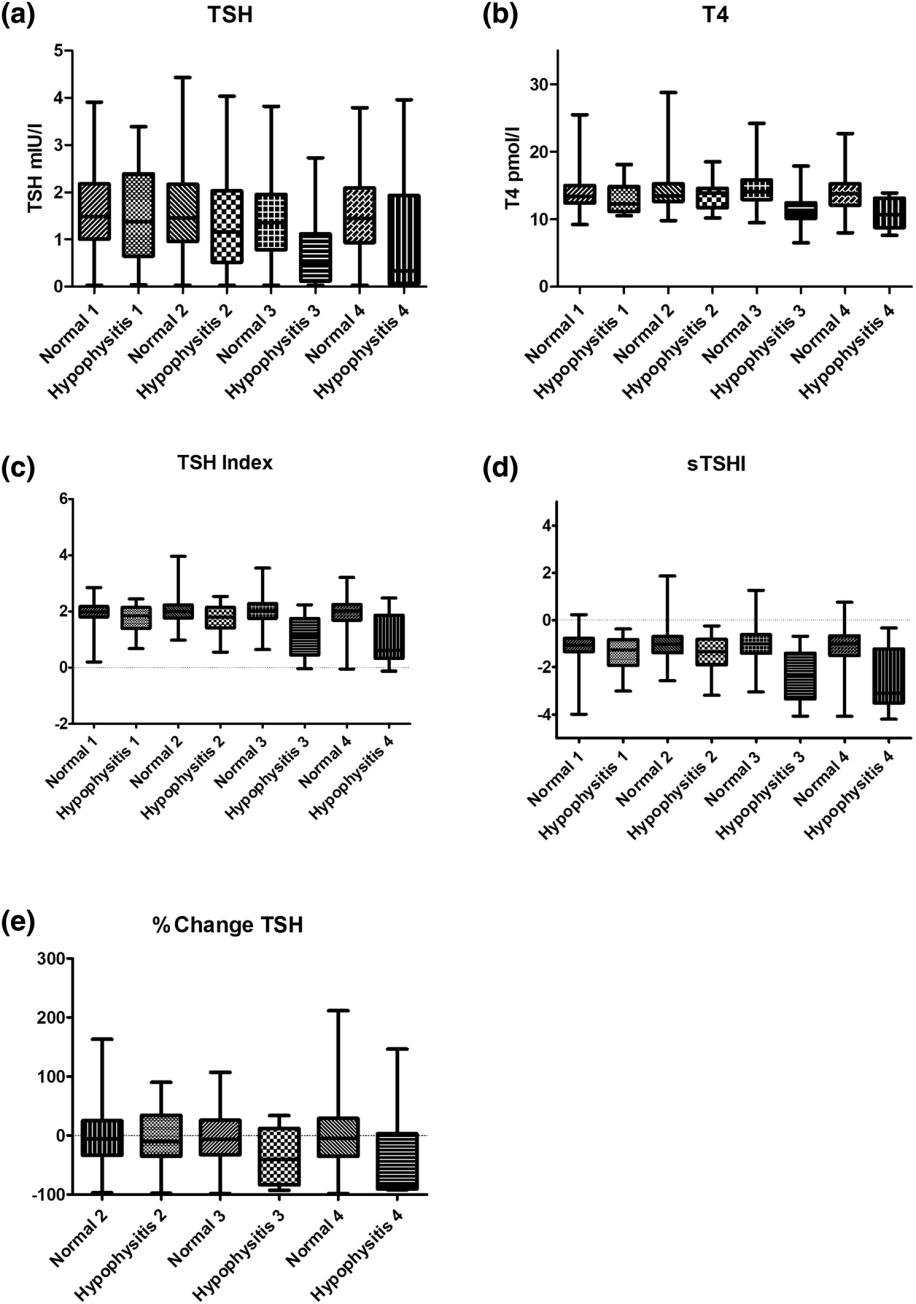
Change in thyroid function over 4 cycles of treatment with ipilimumab in those diagnosed with hypophysitis (left hand columns) and normal controls without hypophysitis (right hand columns). **a** TSH **b** Free T4 **c** TSH index **d** standardised TSH index and **e** % change in TSH from baseline (pre-cycle 1). All graphs show whisker plots with vertical bars indicating minimum and maximum values. Number if included patients at each cycle: Hypophysits Cycle 1 = 24, Cycle 2 = 19, Cycle 3 = 17 and Cycle 4 = 10; Controls Cycle 1 = 217, Cycle 2 = 168, Cycle 3 = 117, Cycle 4 = 86

In patients who developed hypophysitis, although there was a trend of declining TSH levels over the first 3 cycles of treatment, there was considerable overlap in TSH levels between different cycles (Fig. 2a). The mean TSH in cycle 4 was higher than the mean TSH in cycle 3; however, this could be attributed to fewer numbers available for analysis. There was no significant difference in TSH between the hypophysitis cohort and controls at cycle 3 and 4.

ROC analysis of TSH for cycle 3 showed a cut off of 0.74 gave a sensitivity of 65% (CI 38 to 86%) with a specificity of 80% (72 to 86%), whilst a cutoff of 2.62 gave a sensitivity of 94% (71 to 99) but with a specificity of only 10% (5 to 17).

**Fig. 3:**
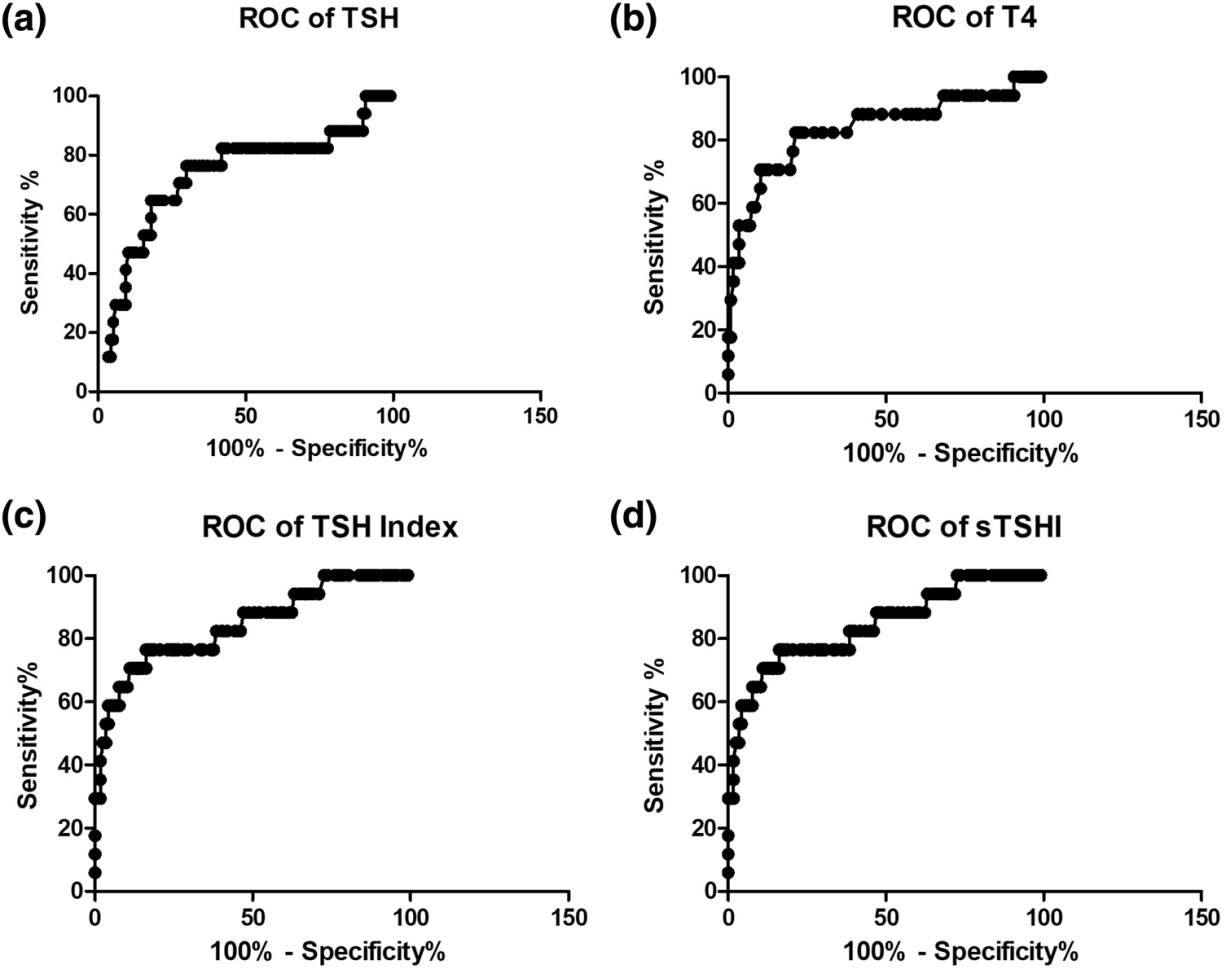
ROC curves showing the diagnostic performance of TSH, Free T4, TSH index and standardised TSH index to distinguish individuals with hypothysitis from those without hypophysitis on the basis of thyroid function tests prior to cycle 3 of ipilimumab

Area under the ROC (AUROC) was 0.732 (CI 0.5859–0.8782) (Fig. 3a).

### Free T4

When comparing the IH and non-IH groups, a significant difference in Free T4 was observed prior to cycle 3 (11.35 pmol/l ± 2.602 vs 14.54 pmol/l ± 2.664, adjusted *P* < 0.001) and cycle 4 (10.76 pmol/l ± 2.326 vs 14.07 pmol/l ± 2.684, adjusted *P* = 0.05) (Fig. 2b).

ROC analysis of Free T4 for cycle 3 demonstrated that a T4 of 12.35 pmol/l gave a sensitivity of 70% (44 to 90) with a specificity of 80% (72 to 87) and a T4 of 15.35 pmol/l gave sensitivity of 94% (71 to 99) but with a specificity of 32% (23 to 41) AUROC was 0.837 at cycle 3 (CI 0.713–0.961) (Fig. 3b).

### TSH index and standardised TSH index

Comparing the TSH index (TSHI) and standardised TSH index (sTSHI) between IH and non-IH groups demonstrated a highly significant difference. At cycle 3 the mean TSH Index was 1.124 in those with hypophysitis and 1.99 in those without (*p* < 0.001), whilst at cycle 4 the mean was 0.952 in those with hypophysitis and 1.95 in those without (*p* < 0.05) (Fig. 2c). ROC analysis at Cycle 3 showed a value of 1.675 gave a sensitivity of 76% (50 to 93) with a specificity of 81% (73 to 88), whilst a level of 2.125 gave a sensitivity of 94% (71 to 99) with s specificity of 37% (28 to 46) The area under the curve was 0.843 (0.730 to 0.956) (Fig. 3c).

The mean sTSH Index at cycle 3 was − 2.332 in those with hypophysitis and 1.049 in those without (*p* < 0.001), whilst at cycle 4 it was − 2.585 in those with hypophysitis and − 1.109 in those without (*p* < 0.05) (Fig. 2d).

sTSHI has similar predictive value on ROC analysis at cycle 3. A value of − 1.515 gave sensitivity of 76% (50 to 93) with specificity of 81% (73 to 88) and − 0.85 gave a sensitivity of 94% (71 to 99) with a specificity of 37% (28 to 46). Area under the curve was 0.842 (0.729 to 0.956). (Fig. 3d).

### Percentage change in TSH

There was no significant difference in the percentage change in TSH from baseline in either group at cycle 2 through 4. (Fig. 2e).

A percentage fall of TSH of 39% gave a sensitivity of 53% (27 to 79) at a specificity of 80% (71 to 87) and a fall of 34% gave a sensitivity of 100% (78 to 100) but with specificity of only 18% (11 to 27). Previous reports have suggested a fall of 80% of TSH to be a useful discriminator to diagnose hypophysitis. In our study this had a sensitivity of only 27% (8 to 55) albeit with a specificity of 93% (86 to 97). Area under the curve for % change in TSH was only 0.667 (0.513 to 0.822) (data not shown).

## Discussion

This study has examined a single centre cohort of patients treated with ipilimumab as monotherapy or in combination with Nivolumab for advanced melanoma, defining all those with hypophysitis through rigorous diagnostic criteria.

In this study, the incidence of hypophysitis in the ipilimumab-treated cohort was 8.1%. This was in line with the literature which reports an incidence of 3.9–13.3% for ipilmumab monotherapy and 7.7–15.2% for combination therapy.

Gender was not a risk factor for developing IH in this study. Although the incidence of hypophysitis was greater in females (10.2%) compared to males (8.2%), this did not reach statistical significance. In contrast, other studies have found that IH is more frequent in males than females [11, 15]. As the size of the hypophysitis cohort in our study is comparable to that of the other two studies, the reason for the discrepancy in incidence between genders is uncertain. The hypophysitis cohort had an older median age of 62 years compared to 56 years for the no hypophysitis cohort, but again this did not reach significance. Faje et al. found older age to be a risk factor for developing IH [11].

The median number of treatment cycles received in the hypophysitis cohort and the no hypophysitis cohort was similar. This likely reflects current clinical practice to continue ipilimumab treatment despite the development of hypophysitis when possible as hypopituitarism resulting from hypophysitis was likely to be irreversible [16].

The majority of patients who developed IH experienced a headache (88.9%). Headache in hypophysitis is likely caused by the expanding pituitary mass distending the dura mater and compressing the sellae [17]. 12 of 24 patients who experienced a headache were found to have an enlarged pituitary gland on MRI. All three patients without headache had a normal pituitary gland on MRI. The time from onset of headache to MRI did not influence the pituitary gland findings on MRI. This study did not determine the incidence of headache in the no hypophysitis cohort for comparison so we cannot comment on the discriminatory value of this symptom. We did not include headache as part of the diagnostic criteria for hypophysitis, in part to ensure the diagnostic criteria were robust. However, it would seem clinically appropriate to evaluate any patient receiving immunotherapy with a headache carefully for the presence of endocrine abnormalities.

No ipilimumab-related visual field defects were recorded which is in line with the literature [11]. Visual abnormalities are rarer in IH compared to autoimmune hypophysitis as the degree of pituitary gland enlargement is usually milder [18].

In terms of endocrine dysfunction, again this is broadly in line with the literature with ACTH deficiency being the most common, followed by thyroid then gonadal deficiency. We identified both high and low prolactin levels at the time of presentation, we hypothesise that elevated prolactin may result from pituitary enlargement and stalk compression. However, neither occurred frequently enough to be reliable to detect hypophysitis.

As recommended in ipilimumab SPC, thyroid functions are usually tested prior to each treatment cycle, and therefore, often, the first endocrine abnormality detected. In a recent study, a fall in TSH > 80% has been suggested as possible early marker of IH but despite being specific at 100%, it was reported to have sensitivity of just 55% [12]. This was not demonstrated in our study, where the declining trend in TSH and % change in TSH prior to cycles 2–4 from TSH before cycle 1 was not statistically significant and there was a large degree of overlap between treatment cycles. ROC analysis demonstrated a specificity of just 10% when TSH cut off below 2.62 mIU/l was used to achieve a sensitivity of 94%. Percentage change in TSH was similarly not useful. The different conclusions could be due to the larger cohort of hypophysitis patients in our study, a more robust case definition for hypophysitis, and differing numbers of patients with secondary hypothyroidism.

On the contrary, a fall in free T4 before treatment cycles 3 and 4 proved to be better in predicting subsequent development of hypophysitis. However, on ROC analysis, the sensitivity was low so it is unclear how useful it would be in clinical practice.

TSH index and standardised TSH indexes have been proposed as a more accurate indicator of secondary hypothyroidism. A significant difference in TSHI and sTSHI between hypophysitis and non hypophysitis cohorts was observed prior to cycles 3 and 4. sTSHI > − 1.06 at cycle 3 had a sensitivity of 83.33% but specificity of 52.8% only (AUC 0.803). We did not analyse the diagnostic performance of these tests at cycle 4 due to the small number of patients.

Secondary adrenal insufficiency was the most common endocrine abnormality detected, and this is also likely to be the most clinically significant. We have not evaluated the value in monitoring cortisol levels to predict hypophysitis, but the diurnal pattern and variability between individuals is likely to make this of limited use.

This study was carried out at a single institution, limiting variance in diagnosis and management. The size of the ipilimumab-treated cohort was also comparable to other studies addressing IH [11, 15]. However, as only thyroid function tests were measured prospectively in all patients, the diagnosis of hypophysitis may have relied on a change in thyroid function or clinical suspicion to prompt a more detailed endocrine and imaging workup, meaning it is possible that mild pituitary enlargement or dysfunction was missed. Notably only 1 patient had secondary hypothyroidism as their sole diagnostic criteria, and they also had post menopausal LH deficiency, suggesting the diagnosis of hypophysitis was robust. It is also important to consider the potential confounding effect of a sick euthyroid status, mild thyroiditis induced by the immunotherpay resulting in low TSH but normal T4 [19], and glucocorticoids supressing TSH [20]. However, these would all have been expected to reduce differences between the groups and, therefore, are unlikely to affect the conclusions.

Although this study focused on ipilimumab, either alone or in combination, the PD-1 inhibitors such as nivolumab or pembrolizumab are also reported to cause pituitary dysfunction, but mostly isolated ACTH deficiency [21]. This can still result in life threatening cortisol deficiency, but thyroid abnormalities when seen are usually the result of primary hyper or hypothyroidism, rather than secondary hypothyroidism. Therefore, changes in thyroid function would not be expected to predict the onset of ACTH deficiency with these drugs. However, it is noteworthy that the combination of ipilimumab and nivolumab is now also licensed for the treatment of renal cancer, and hence the numbers of patients at risk of hypophysitis is likely to increase [22].

In conclusion, TSH is not useful in predicting evolving hypophysitis, and therefore, frequent monitoring is not recommended to screen for IH. Free T4, TSH index and standardised TSH index may be valuable but with limited sensitivity and specificity. Therefore, a high index of clinical suspicion remains paramount in early detection of hypophysitis. Further studies are warranted to identify better markers of incipient hypophysitis, to avoid patients presenting with the consequences of hypopituitarism.

## Electronic supplementary material

Below is the link to the electronic supplementary material.

Supplementary file1 (DOCX 23 kb)

Supplementary file2 (DOCX 37 kb)

## References

[1] Manola J, Atkins M, Ibrahim J, Kirkwood J (2000). Prognostic factors in metastatic melanoma: a pooled analysis of Eastern Cooperative Oncology Group trials. J Clin Oncol.

[2] Wolchok JD, Hodi FS, Weber JS, Allison JP, Urba WJ, Robert C, O'Day SJ, Hoos A, Humphrey R, Berman DM, Lonberg N, Korman AJ (2013). Development of ipilimumab: a novel immunotherapeutic approach for the treatment of advanced melanoma. Ann N Y Acad Sci.

[3] Weber JS, D'Angelo SP, Minor D, Hodi FS, Gutzmer R, Neyns B, Hoeller C, Khushalani NI, Miller WH, Lao CD, Linette GP, Thomas L, Lorigan P, Grossmann KF, Hassel JC, Maio M, Sznol M, Ascierto PA, Mohr P, Chmielowski B, Bryce A, Svane IM, Grob JJ, Krackhardt AM, Horak C, Lambert A, Yang AS, Larkin J (2015). Nivolumab versus chemotherapy in patients with advanced melanoma who progressed after anti-CTLA-4 treatment (CheckMate 037): a randomised, controlled, open-label, phase 3 trial. Lancet Oncol.

[4] Postow MA, Chesney J, Pavlick AC, Robert C, Grossmann K, McDermott D, Linette GP, Meyer N, Giguere JK, Agarwala SS, Shaheen M, Ernstoff MS, Minor D, Salama AK, Taylor M, Ott PA, Rollin LM, Horak C, Gagnier P, Wolchok JD, Hodi FS (2015). Nivolumab and ipilimumab versus ipilimumab in untreated melanoma. N Engl J Med.

[5] Wolchok JD, Rollin L, Larkin J (2017). Nivolumab and ipilimumab in advanced melanoma. N Engl J Med.

[6] Kumar V, Chaudhary N, Garg M, Floudas CS, Soni P, Chandra AB (2017). Current diagnosis and management of immune related adverse events (irAEs) induced by immune checkpoint inhibitor therapy. Front Pharmacol.

[7] Barroso-Sousa R, Barry WT, Garrido-Castro AC, Hodi FS, Min L, Krop IE, Tolaney SM (2017). Incidence of endocrine dysfunction following the use of different immune checkpoint inhibitor regimens: a systematic review and meta-analysis. JAMA Oncol.

[8] Albarel F, Gaudy C, Castinetti F, Carré T, Morange I, Conte-Devolx B, Grob J-J, Brue T (2015). Long-term follow-up of ipilimumab-induced hypophysitis, a common adverse event of the anti-CTLA-4 antibody in melanoma. Eur J Endocrinol.

[9] Snyders T, Chakos D, Swami U, Latour E, Chen Y, Fleseriu M, Milhem M, Zakharia Y, Zahr R (2019). Ipilimumab-induced hypophysitis, a single academic center experience. Pituitary.

[10] Iwama S, De Remigis A, Callahan MK, Slovin SF, Wolchok JD, Caturegli P (2014). Pituitary expression of CTLA-4 mediates hypophysitis secondary to administration of CTLA-4 blocking antibody. Sci Transl Med.

[11] Faje AT, Sullivan R, Lawrence D, Tritos NA, Fadden R, Klibanski A, Nachtigall L (2014). Ipilimumab-induced hypophysitis: a detailed longitudinal analysis in a large cohort of patients with metastatic melanoma. J Clin Endocrinol Metab.

[12] De Sousa SMC, Sheriff N, Tran CH, Menzies AM, Tsang VHM, Long GV, Tonks KTT (2018). Fall in thyroid stimulating hormone (TSH) may be an early marker of ipilimumab-induced hypophysitis. Pituitary.

[13] Jostel A, Ryder WDJ, Shalet SM (2009). The use of thyroid function tests in the diagnosis of hypopituitarism: definition and evaluation of the TSH Index. Clin Endocrinol.

[14] Ryder M, Callahan M, Postow MA, Wolchok J, Fagin JA (2014). Endocrine-related adverse events following ipilimumab in patients with advanced melanoma: a comprehensive retrospective review from a single institution. Endocr Relat Cancer.

[15] Min L, Hodi FS, Giobbie-Hurder A, Ott PA, Luke JJ, Donahue H, Davis M, Carroll RS, Kaiser UB (2015). Systemic high-dose corticosteroid treatment does not improve the outcome of ipilimumab-related hypophysitis: a retrospective cohort study. Clin Cancer Res.

[16] Larkin J, Chiarion-Sileni V, Gonzalez R, Grob JJ, Cowey CL, Lao CD, Schadendorf D, Dummer R, Smylie M, Rutkowski P, Ferrucci PF, Hill A, Wagstaff J, Carlino MS, Haanen JB, Maio M, Marquez-Rodas I, McArthur GA, Ascierto PA, Long GV, Callahan MK, Postow MA, Grossmann K, Sznol M, Dreno B, Bastholt L, Yang A, Rollin LM, Horak C, Hodi FS, Wolchok JD (2015). Combined nivolumab and ipilimumab or monotherapy in untreated melanoma. N Engl J Med.

[17] Torino F, Barnabei A, De Vecchis L, Salvatori R, Corsello SM (2012). Hypophysitis induced by monoclonal antibodies to cytotoxic T lymphocyte antigen 4: challenges from a new cause of a rare disease. Oncologist.

[18] Faje A (2016). Immunotherapy and hypophysitis: clinical presentation, treatment, and biologic insights. Pituitary.

[19] Morganstein DL, Lai Z, Spain L, Diem S, Levine D, Mace C, Gore M, Larkin J (2017). Thyroid abnormalities following the use of cytotoxic T-lymphocyte antigen-4 and programmed death receptor protein-1 inhibitors in the treatment of melanoma. Clin Endocrinol.

[20] Beck-Peccoz P, Rodari G, Giavoli C, Lania A (2017). Central hypothyroidism—a neglected thyroid disorder. Nat Rev Endocrinol.

[21] Ariyasu R, Horiike A, Yoshizawa T, Dotsu Y, Koyama J, Saiki M, Sonoda T, Nishikawa S, Kitazono S, Yanagitani N, Nishio M (2017). Adrenal insufficiency related to anti-programmed death-1 therapy. Anticancer Res.

[22] Motzer RJ, Tannir NM, McDermott DF, Arén Frontera O, Melichar B, Choueiri TK, Plimack ER, Barthélémy P, Porta C, George S, Powles T, Donskov F, Neiman V, Kollmannsberger CK, Salman P, Gurney H, Hawkins R, Ravaud A, Grimm M-O, Bracarda S, Barrios CH, Tomita Y, Castellano D, Rini BI, Chen AC, Mekan S, McHenry MB, Wind-Rotolo M, Doan J, Sharma P, Hammers HJ, Escudier B (2018). Nivolumab plus ipilimumab versus sunitinib in advanced renal-cell carcinoma. N Engl J Med.

