# Supplementary material for: Predicting development of ipilimumab-induced hypophysitis: utility of T4 and TSH index but not TSH
Source: J Endocrinol Invest. 2020 May 24;44(1):195–203. doi: 10.1007/s40618-020-01297-3 (PMC7796881; doi:10.1007/s40618-020-01297-3)
Supplement: Supplementary file 1 — Supplementary file1 (DOCX 23 kb) [file 40618_2020_1297_MOESM1_ESM.docx]

| \| Patient Number \| Gender \| Age  (Years) \| Treatment received \| Headache  (Yes/No) \| Fatigue  (Yes/No) \| Head MRI finding \| Onset of hypophysitis \| Endocrine dysfunctions \| \| --- \| --- \| --- \| --- \| --- \| --- \| --- \| --- \| --- \| \| 1 \| Female \| 73 \| Ipilimumab \| No \| Yes \| Normal pituitary gland \| After 1 cycle \| Secondary adrenal insufficiency \| \| 2 \| Male \| 75 \| Ipilimumab \| Yes \| Yes \| Enlarged pituitary gland \| After 2 cycles \| Secondary hypothyroidism  Secondary adrenal insufficiency  Secondary hypogonadism  Hypoprolactinemia \| \| 3 \| Male \| 50 \| Ipilimumab \| Yes \| Yes \| Normal pituitary gland \| After 3 cycles \| Secondary Hypothyroidism  Secondary hypogonadism  Secondary adrenal insufficiency  Hypoprolactinemia \| \| 4 \| Male \| 73 \| Ipilimumab \| Yes \| No \| Enlarged pituitary gland \| After 3 cycles \| Secondary hypothyroidism  Secondary adrenal insufficiency  Hypoprolactinemia \| \| 5 \| Male \| 67 \| Ipilimumab \| Yes \| No \| Normal pituitary gland \| After 3 cycles \| Secondary hypothyroidism  Secondary adrenal insufficiency \| \| 6 \| Male \| 67 \| Ipilimumab \| Yes \| Yes \| Enlarged pituitary gland \| After 4 cycles \| Secondary hypothyroidism  Secondary hypogonadism  Secondary adrenal insufficiency  Hypoprolactinemia \| \| 7 \| Male \| 47 \| Ipilimumab \| Yes \| Yes \| Normal pituitary gland \| After 2 cycles \| Secondary adrenal insufficiency \| \| 8 \| Female \| 35 \| Ipilimumab \| Yes \| Yes \| Enlarged pituitary gland \| After 3 cycles \| Secondary adrenal insufficiency  Secondary hypothyroidism  Secondary Hypogonadism \| \| 9 \| Male \| 66 \| Ipilimumab \| Yes \| Yes \| Normal pituitary gland \| After 2 cycles \| Secondary Hypothyroidism  Secondary adrenal insufficiency  Secondary hypogonadism  Hypoprolactinemia \| \| 10 \| Male \| 67 \| Ipilimumab \| Yes \| Yes \| Normal pituitary gland \| After 4 cycles \| Secondary hypothyroidism  Secondary hypogonadism  Secondary adrenal insufficiency  Hypoprolactinemia \| \| 11 \| Female \| 79 \| Ipilimumab \| No \| Yes \| Normal pituitary gland \| After 4 cycles \| Secondary adrenal insufficiency *  LH deficiency (post menopausal) \| \| 12 \| Male \| 32 \| Ipilimumab \| Yes \| Yes \| Normal pituitary gland \| After 4 cycles \| Secondary hypothyroidism  Secondary adrenal insufficiency  Secondary hypogonadism  Hypoprolactinemia \| \| 13 \| Female \| 65 \| Ipilimumab \| Yes \| Yes \| Normal pituitary gland \| After 3 cycles \| Secondary hypothyroidism  Secondary adrenal insufficiency  LH and FSH deficiency (post menopausal)  Hypoprolactinemia \| \| 14 \| Female \| 60 \| Ipilimumab \| Yes \| Yes \| Enlarged pituitary gland \| After 1 cycle \| Secondary hypothyroidism  Secondary adrenal insufficiency  LH deficiency (post menopausal)  Hypoprolactinemia \| \| 15 \| Female \| 51 \| Ipilimumab \| Yes \| Yes \| Normal pituitary gland \| After 2 cycles \| Secondary adrenal insufficiency  Hypoprolactinemia \| \| 16 \| Male \| 70 \| Ipilimumab \| Yes \| No \| Enlarged pituitary gland \| After 3 cycles \| Secondary hypothyroidism  Secondary adrenal insufficiency \| \| 17 \| Female \| 65 \| Ipilimumab \| Yes \| Yes \| Enlarged pituitary gland \| After 3 cycles \| Secondary hypothyroidism  Secondary adrenal insufficiency  LH and FSH deficiency (post menopausal)  Hypoprolactinemia \| \| 18 \| Female \| 68 \| Ipilimumab \| Yes \| No \| Enlarged pituitary gland \| After 2 cycles \| Secondary hypothyroidism  Secondary adrenal insufficiency  LH deficiency (post menopausal) \| \| 19 \| Female \| 60 \| Ipilimumab and Nivolumab \| Yes \| Yes \| Enlarged pituitary gland \| After 2 cycles \| Secondary adrenal insufficiency \| \| 20 \| Female \| 67 \| Ipilimumab and Nivolumab \| No \| Yes \| Normal pituitary gland \| After 4 cycles \| Primary subclinical hypothyroidism  Secondary adrenal insufficiency  Hyperprolactinemia \| \| 21 \| Male \| 59 \| Ipilimumab \| Yes \| Yes \| Not done \| After 2 cycles \| Secondary hypothyroidism  Secondary adrenal insufficiency  Secondary hypogonadism  Hypoprolactinemia \| \| 22 \| Male \| 62 \| Ipilimumab \| Yes \| Yes \| Enlarged pituitary gland \| After 4 cycles \| Secondary adrenal insufficiency  Secondary hypothyroidism \| \| 23 \| Female \| 57 \| Ipilimumab \| Yes \| Yes \| Enlarged pituitary gland \| After 2 cycles \| Secondary adrenal insufficiency *  LH and FSH deficiency (post menopausal) \| \| 24 \| Female \| 65 \| Ipilimumab \| Yes \| Yes \| Normal pituitary gland \| After 3 cycles \| Secondary hypothyroidism  LH deficiency (post menopausal) \| \| 25 \| Male \| 77 \| Ipilimumab and Nivolumab \| Yes \| Yes \| Enlarged pituitary gland \| After 3 cycles \| Secondary adrenal insufficiency  ** \| |
| --- | --- | --- | --- | --- | --- | --- | --- | --- | --- | --- | --- | --- | --- | --- | --- | --- | --- | --- | --- | --- | --- | --- | --- | --- | --- | --- | --- | --- | --- | --- | --- | --- | --- | --- | --- | --- | --- | --- | --- | --- | --- | --- | --- | --- | --- | --- | --- | --- | --- | --- | --- | --- | --- | --- | --- | --- | --- | --- | --- | --- | --- | --- | --- | --- | --- | --- | --- | --- | --- | --- | --- | --- | --- | --- | --- | --- | --- | --- | --- | --- | --- | --- | --- | --- | --- | --- | --- | --- | --- | --- | --- | --- | --- | --- | --- | --- | --- | --- | --- | --- | --- | --- | --- | --- | --- | --- | --- | --- | --- | --- | --- | --- | --- | --- | --- | --- | --- | --- | --- | --- | --- | --- | --- | --- | --- | --- | --- | --- | --- | --- | --- | --- | --- | --- | --- | --- | --- | --- | --- | --- | --- | --- | --- | --- | --- | --- | --- | --- | --- | --- | --- | --- | --- | --- | --- | --- | --- | --- | --- | --- | --- | --- | --- | --- | --- | --- | --- | --- | --- | --- | --- | --- | --- | --- | --- | --- | --- | --- | --- | --- | --- | --- | --- | --- | --- | --- | --- | --- | --- | --- | --- | --- | --- | --- | --- | --- | --- | --- | --- | --- | --- | --- | --- | --- | --- | --- | --- | --- | --- | --- | --- | --- | --- | --- | --- | --- | --- | --- | --- | --- | --- | --- | --- | --- | --- | --- | --- | --- | --- | --- | --- | --- | --- | --- |

* -received thyroxine on clinical grounds with falling T4 but did not meet diagnostic criteria for secondary hypothyroidism

**- transient low testosterone on finasteride therefore not diagnosed as secondary hypogonadism
