# Supplementary material for: Predicting development of ipilimumab-induced hypophysitis: utility of T4 and TSH index but not TSH
Source: J Endocrinol Invest. 2020 May 24;44(1):195–203. doi: 10.1007/s40618-020-01297-3 (PMC7796881; doi:10.1007/s40618-020-01297-3)
Supplement: Supplementary file 2 — Supplementary file2 (DOCX 37 kb) [file 40618_2020_1297_MOESM2_ESM.docx]

Supplementary Figure 1. Summary of included and excluded patients

66 were excluded

49 did not have baseline thyroid functions

17 developed rise in TSH above range

100 received less than 3 cycles

8 were excluded

1 did not have baseline thyroid functions

7 developed hypophysitis before Cycle 3

17 included in thyroid function analysis

117 included in thyroid function analysis

25 developed hypophysitis

283 did not develop hypophysitis

308 patients were identified

277 received Ipilimumab

31 received Ipilimumab + Nivolumab
